# Glomerulonephritis Pattern at a Jordanian Tertiary Care Center

**DOI:** 10.1155/2018/2751372

**Published:** 2018-10-11

**Authors:** Randa I. Farah

**Affiliations:** Department of Internal Medicine, School of Medicine, University of Jordan, Amman, Jordan

## Abstract

**Aim:**

To determine the prevalence and frequency of different pathological patterns of glomerulonephritis (GN) in adolescent (age ≥ 11 years) and adult Jordanian patients.

**Materials and Methods:**

A retrospective analysis of all clinical and pathological reports of Jordanian patients who had native renal biopsies at the University of Jordan hospital between January 2007 and March 2018 to assess the prevalence and pathological pattern of GN. The data were analyzed statistically using descriptive statistics, the chi-squared test, and Fisher's exact tests. The level of significance was set at *P* < 0.05.

**Results:**

Two hundred and nine patients (88 males and 121 females) had native kidney biopsies diagnosed as having GN; the mean age at the time of biopsy was 36.0 ± 14.9 years. Primary GN (51.2%) was more common than secondary GN (48.8%). The most common GN was lupus nephritis (LN) (33.5%), followed by membranous nephropathy (MGN) (15.3%), and diabetic nephropathy (DN) (11.0%). Furthermore, IgA nephropathy was noted in 8.1% of cases. LN was the most common among the secondary GN and occurred in 49.6% of females; MGN was the most common primary GN and occurred in 22.7% of males. There was a statistically significant difference between males and females in the prevalence of LN and MGN (*P* < .001 and *P* = .011, respectively). LN was also dominant in all age groups expect for the ≥60 years group, which tended to exhibit DN (40%).

**Conclusion:**

LN is the most common GN type in Jordan, followed by MGN and DN. MGN is the predominant primary GN with a higher prevalence among males; LN is the predominant secondary GN and tends to occur in Jordanian females. The GN patterns in this study shifted from membranoproliferative GN to MGN in Jordan, which revealed a shift towards similar patterns exhibited in developed countries. Furthermore, DN is the most frequent GN in the elderly.

## 1. Introduction

Glomerulonephritis (GN) is a generic term for glomeruli injuries, which may range from massive inflammatory injuries that largely destroy the glomerulus to the injuries that can only be detected using sensitive techniques such as electron microscopy. GN is one of the main causes of chronic kidney disease and end-stage renal disease (ESRD) worldwide, which are responsible for major morbidity and mortality worldwide [1]. To establish a definitive diagnosis of GN and specify the pattern of glomerular injury, a histopathological and immunohistochemical analysis of the tissues typically obtained from patient's kidney via renal biopsy is necessary. Each type of GN has a specific histopathological pattern but does not have a specific clinical entity [2].

Glomerulonephritis can be classified into primary glomerular disease (primary GN) when there is no associated disease and secondary glomerular disease (secondary GN) when glomeruli involvement is part of a systemic disease such as connective tissue diseases, systemic lupus erythematosus, systemic vasculitis, and infective endocarditis. However, the mechanisms of glomerular injury are still not fully understood [3].

The incidence and period prevalence of GN resulting in death and ESRD in Jordan are unknown. A careful study of the incidence of different histopathological patterns of GN is necessary to establish the patterns and trends of glomerular diseases in a specific geographical area [4]. This will improve our ability to monitor disease trends, to formulate policies for an early detection of disease, and to take appropriate preventive and interventional measures.

Unfortunately, there are no recent data on the incidence of different histopathological patterns of GN in Jordan. Therefore, the aim of this cross-sectional study was to determine the prevalence and incidence of different histopathological patterns of GN in adolescent and adult patients in Jordan.

## 2. Materials and Methods

The pathological reports of native kidney biopsies, completed between January 2007 and March 2018, of 315 patients aged >11 years were retrieved from digital archives of the pathology department of Jordan University Hospital and were reviewed retrospectively. Indications for kidney biopsy were nephrotic proteinuria, nonnephrotic proteinuria with or without hematuria, unexplained acute kidney injury, unexplained chronic kidney disease, isolated glomerular hematuria, and systemic lupus erythromatous with evidence of renal involvement. All biopsies had been conducted percutaneously using real-time ultrasound guidance by a nephrologist or interventional radiologist. The specimens were prepared and examined in the hospital's pathological laboratory using light microscopy and immunofluorescence techniques. A few samples were processed (fixed and stained) and examined under electron microscopy.

The reports of kidney transplant biopsies, in addition to biopsies that revealed nonglomerular pathologies, including renal malignancies, tubulointerstitial diseases, and those with “no detectable abnormality” or “non-specific changes,” were excluded from the study. The pathological reports of the remaining 209 patients with GN-diagnosed biopsies were investigated further, and their medical records, including biochemistry and urinalysis results, histopathological findings, and clinical indications of biopsy, were collected. The clinical indication was reported. The demographic information including patient sex, age at the time of biopsy, and nationality was also recorded. Repeat biopsies for the same patients during the study period were considered if they had a different clinical indication or pathological finding. This study was conducted in accordance with the World Medical Association Declaration of Helsinki principles, and ethical approval was obtained as per the university hospital protocols.

### 2.1. Statistical Analysis

All analyses were performed using SPSS version 20 (IBM Corp., Chicago, IL, USA). *P* values less than 0.05 indicated statistical significance. Data are presented as means, standard deviations, frequencies, and percentages. We used the chi-squared test for association or Fisher's exact test (when the expected cell frequencies were less than 5) to investigate the statistically significant relationships between patients' sex and different histopathological patterns of GN.

### 2.2. Results

**Figure 1:**
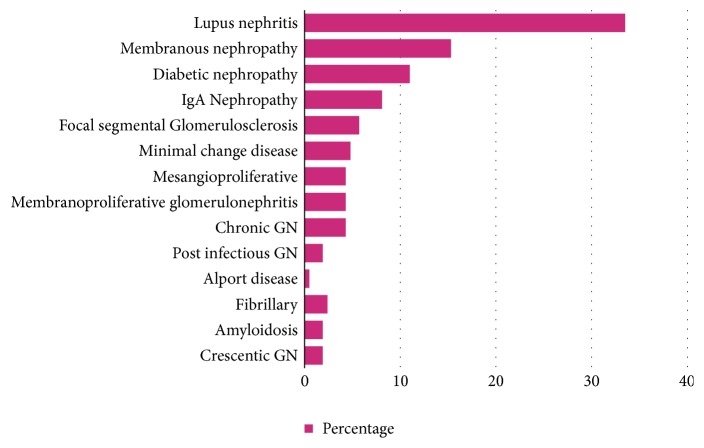
Incidence of different histopathological patterns of glomerulonephritis in the study population.

The records of native kidney biopsies of 209 patients were included and analyzed in this study. The mean age of patients at the time of biopsy was 36.0 ± 14.9 years (range: 11–77 years); 88 (42.11%) patients in the cohort were male, and 121 (57.9%) were female. One hundred and seventy-one patients (81.8%) were aged 19–59 years, 23 (11.0%) patients were aged ≤18 years, and 15 (7.2%) patients were aged ≥60 years. There were 107 patients (51.2%) with primary GN and 102 (48.8%) patients with secondary GN. The most common type of GN was lupus nephritis (33.5%), followed by membranous nephropathy (15.3%), and diabetic nephropathy (11.0%); the least common types of GN were Alport syndrome with an incidence of less than 1% and amyloidosis, crescentic GN, and postinfectious GN with an incidence of 4 (1.9%) each (Table 1, Figure 1). The four cases of crescentic GN were reported within the primary GN supgroup because there were no signs of involvement by immunological or other systemic disease in these cases.

Roughly three quarters (74.5%) of patients who presented secondary GN were females; 57.9% of primary GN biopsies were from males (Table 2). There was a statistically significant association between patients' sex and histopathological presentation of GN (primary or secondary) (*χ*^2^ (1) = 22.563, *P* < .001) with a moderate strength association (*φ* = 0.329, *P* < .001). The most common type of GN in male patients was membranous nephropathy (22.7%) followed by diabetic nephropathy (12.5%). In female patients, the most common type was lupus nephritis (49.6%) followed by membranous nephropathy and diabetic nephropathy (9.9% each). There was a statistically significant difference between male and female patients in terms of the prevalence of lupus nephritis (*P* < 0.001; Table 1).

Lupus nephritis was the most common histopathological pattern in young patients aged ≤18 years (43.5%) and in patients aged 19–59 years (33.9%). Diabetic nephropathy was the most common histopathological pattern in patients older than 60 years (40.0%; Table 3).

**Figure 2:**
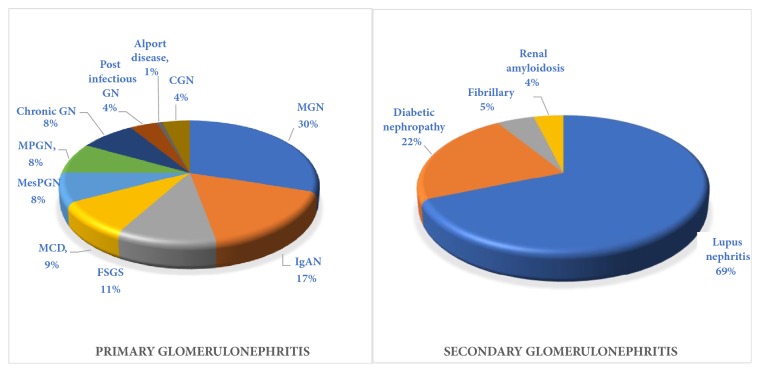
Different histopathological patterns of glomerulonephritis within the subgroup of primary and secondary GN.

Lupus nephritis was the most common among secondary GN (68.6%), followed by diabetic nephropathy (22.5%); membranous nephropathy was the most common primary GN (30.0%), followed by IgA nephropathy (16.6%) (Figure 2).

### 2.3. Discussion

In this study, I reported the incidence of different histopathological patterns of GN in an adult patient population at Jordan University Hospital. This hospital is a tertiary care university-based hospital in the center of Amman, Jordan, with a capacity of approximately 600 beds [5]. It receives a large number of patients from all parts of Jordan, thus providing a good representative sample for our study, and all subjects in our study were Jordanians. I studied the incidence of different histopathological patterns of GN in Jordan between January 2007 and March 2018 and the changes in glomerular disease distribution that were associated with patients' age and sex. We compared our results with similar data reported previously from Jordan and other countries.

The prevalence of GN varies worldwide with time and location depending on the genetic profile and environmental exposure of populations [6]. Assessing changes of the glomerular disease pattern is important for an optimal allocation of resources and to focus our research on improving disease outcomes in the future.

Jordan has one of the youngest populations in the world; people aged under 55 years constitute 92% of the population, and the median age is 22.5 years [7]. Most of our patients were younger than 59 years (92%) with a median age of 36 years, and the proportion of elderly patients was quite low (7.5%).

Three studies reporting different histopathological patterns of glomerular disease and representing three different decades have been reported in Jordan [8–10]. The first of these was published by Ghnaimat et al. in 1999, which analyzed the biopsy reports of 191 adult patients from 1994 to 1997. In this study, membranoproliferative GN (MPGN) was the most common histopathological pattern of glomerular disease that was reported in 25% of the patients, and the second most common type was focal segmental glomerulosclerosis (FSGS) that was reported in 22% of the patients [10]. The second study published by Said R et al. in 2000 analyzed the biopsy reports of 350 patients during two periods, i.e., from 1986 to 1989 and from 1997 to 1999. According to this study, MPGN was the most common type of primary glomerular disease that was reported in 18% of the patients, and FSGS was the second most common type reported in 13% of the patients [9]. The latest report was published by Wahbeh et al. in 2008 that included data on 64 patients from 2002 to 2006. According to this study, lupus nephritis was the most common type of glomerular disease reported in 26.5% of the patients, and FSGS was the second most common type of primary glomerular disease reported in 17.2% of the patients [8].

In this cross-sectional study, data were collected for 209 patients from 2007 to 2018. Lupus nephritis was the most common type of glomerular disease reported in 33.5% of the patients; membranous nephropathy was the most commonly observed primary glomerular lesion reported in 15.3% of the patients and 30% of the patients with primary GN (Figure 2); and MPGN was reported in 4.3% of all patients with GN.

Although biopsy-based estimates are subject to selection bias due to clinical criteria used for kidney biopsy indication, the difference between our study and previous three studies may reflect the actual change in histopathological patterns of glomerular diseases. Jordan has changed in terms of social, environmental, and other factors including changes in living and other conditions. These changes may explain the differences in histopathological patterns of glomerular diseases over a period of time [6].

Membranous nephropathy has a high prevalence in Western countries, and there has been a rise in its incidence in different countries [6]. According to this study, changes in the patterns of primary GN (membranous nephropathy is the most common) can be attributed to social and economic factors as well as other factors such as better housing facility, higher standard of living, reduced exposure to infections, and improved healthcare [1]. These factors are also responsible for changing trends in Jordan towards being a more developed and industrialized country [7].

Lupus nephritis is the most common glomerular disease in Jordan. Among all types of GN, lupus nephritis has the highest incidence at 33.5% of the GN cases in Jordan (68.6% of the cases with secondary GN) and is considered to have the highest prevalence in the age group of 19-59 years, with a higher prevalence in female patients (*P* < 0.05) and a male/female ratio of around 1:6; these data are similar to those reported in the United States of America [11]. Contrary to the earlier studies, there is a trend towards an increase in the incidence of lupus nephritis over decades. Ghnaimat et al. and Said et al. [9, 10] reported low incidences of lupus nephritis in their studies at 9.4% and 8%, retrospectively. On the contrary, Wahbeh's [8] and our studies showed a high incidence of lupus nephritis at 26.5% and 33.5%, retrospectively. This can be related to a complex interplay of genetic, hormonal, environmental, and socioeconomic factors [12, 13].

The high incidence of lupus nephritis has been reported in most biopsy-based studies [14, 15]. In the countries neighboring Jordan, lupus nephritis is the most common type of secondary GN, and it is most likely related to the same environmental and genetic factors. For instance, in Oman, lupus nephritis affects 36.15% of the GN cases [13]; in Bahrain, 38.9% of the secondary GN cases [16]; in Egypt, 28.57% of the secondary GN cases [17]; and in Kuwait, 23.4% of secondary GN. The estimated total incidence of lupus nephritis as per the Saudi Arabia registry in 2000 had reached 57% of the secondary GN cases [18].

In this study, diabetic nephropathy was the second most common type of secondary GN reported in 22.5% of the secondary GN cases and 11% of the GN cases, and this is compatible with the data related to the high prevalence of diabetes in Jordan [19] and other countries; however, it may cause an underestimated true incidence of diabetic nephropathy in our population, especially because kidney biopsies are performed in patients with diabetes only if there is suspicion of nephropathy or if there is clinical indication of kidney biopsy. As noted in our study, the high-grade proteinuria with or without renal impairment was the clinical indication of kidney biopsy. Among the neighboring countries of Jordan, Qatar has a diabetic nephropathy prevalence of 50% of secondary GN [20], and Dubai has a diabetic nephropathy prevalence of 14% of secondary GN and 4.4% of GN [21].

MPGN and mesangial proliferative GN were each reported in 4.3% of our patients, and these were less prevalent in our cohort compared with that in earlier studies [8–10] owing to an improved infection control; similar results have been reported in recent data from Saudi Arabia [22] and Dubai [21].

FSGS was reported as the predominant pattern of GN in a previous report from Jordan [8]; however, it was less prevalent in our study. It is still the most common type of primary GN in Saudi Arabia [22] and Iran [23] and the second most common type of primary GN in the United States of America [11]. FSGS prevalence was underestimated in our study because of the clinical indications of kidney biopsy, changes in nephrologist's practice with advanced renal impairment in the presence of proteinuria and hypertension, and absence of renal disease screening strategies.

The incidence of immunoglobulin A nephropathy (IgAN) was slightly higher in our cohort compared to that in earlier studies. It was the second most common type of primary GN, reported in 8.1% of all cases and 18.2% of the primary GN cases [8–10]. IgAN is regarded as the most common type of GN in the world and is most prevalent in Asia (30–40%) and relatively less prevalent in Europe (20%) and North America (10%) [11]; however, it still has a much lower prevalence than that in other countries. These changes may be influenced by clinical indication of kidney biopsy and changes in nephrologist's practice in regard to performing kidney biopsy.

The prevalence of membranous nephropathy in our study was almost twice (6.3% vs. 15.3%) that reported in the earlier studies in Jordan [8], and it was significantly higher in male patients (*P* < 0.05) with a male/female ratio of 1.7:1, which is consistent with its typical racial distribution. All cases of membranous nephropathy in this study were idiopathic with a median age of 38 years. Membranous nephropathy is the most common type of primary GN in Western Saudi Arabia and has a high prevalence in countries such as Iran [23], Italy [24], and the United Arab Emirates [6].

In this study, the incidence of primary GN was slightly more than that of secondary GN (51.2% vs. 48.8%), which is consistent with the global reports and previous reports from Jordan [8–10], but there was a slightly higher incidence of secondary GN compared to that reported in previous studies in Jordan and the neighboring countries. This may be related to a significant increase in the incidence of lupus nephritis and diabetic nephropathy in our population.

The changing patterns of GN in Jordan could be related to the economic changes in the population as 91.0% of the total population is urban, and the annual rate of urbanization is reported as 2.43% [7]. This trend reflects improvement in housing facilities and standards of living with more frequent vaccination and less exposure to infections.

This study had a few limitations. First, this was a single-center study that lacked the data on Jordanian registry for GN. Second, the sample size was limited, which made it difficult to interpret the age- and sex-related differences in several subgroups of this study. Therefore, we recommend that a multicenter study with a larger sample size should be conducted to allow a more comprehensive interpretation of our results among the different subgroups of GN.

## 3. Conclusion

The data presented in this study will help to shed light on the epidemiology of GN in Jordan in the past 10 years. Our results show that lupus nephritis is the most common GN type in Jordan followed by membranous nephropathy and diabetic nephropathy. Membranous nephropathy is the most common type of primary GN with a higher prevalence in the male patient population. Lupus nephritis is the most common type of secondary GN with a higher prevalence in the female patient population. Secondary GN is slightly less common than primary GN. Furthermore, diabetic nephropathy is the most common type of GN in the elderly. The histopathological patterns of GN in this study shifted from MPGN to membranous nephropathy. These results differ notably from those of previous studies conducted in Jordan and reveal a shift towards similar patterns exhibited in developed countries, which indicate that there are fewer cases of GN associated with infections and more cases of GN associated with autoimmune diseases, antigen exposure, ageing, and obesity.

## Figures and Tables

**Table 1:** Distribution of glomerular diseases based on incidence and patients' sex.

**Diagnosis**	**Number (**%**)**	**Male (**%**)**	**Female (**%**)**	**P value**
**Primary Glomerulonephritis**				

MGN	32 (15.3%)	20 (22.7%)	12 (9.9%)	0.018^*∗*^
IgAN	17 (8.1%)	9 (10.2%)	8 (6.6%)	0.244
FSGS	12 (5.7%)	8 (9.1%)	4 (3.3%)	0.071
MCD	10 (4.8%)	6 (6.8%)	4 (3.3%)	0.198
MesPGN	9 (4.3%)	5 (5.7%)	4 (3.3%)	0.309
MPGN	9 (4.3%)	2 (2.3%)	7 (5.8%)	0.189
Chronic GN	9 (4.3%)	7 (8.0%)	2 (1.7%)	0.189
Post infectious GN	4 (1.9%)	3 (3.4%)	1 (0.8%)	0.202
Alport disease	1 (0.5%)	1 (1.1%)	0	0
CGN	4 (1.9%)	2 (2.3%)	2 (1.7%)	1

**Secondary Glomerulonephritis**				

Lupus nephritis	70 (33.5%)	10 (11.4%)	60 (49.6%)	< .001^*∗*^
Diabetic nephropathy	23 (11.0%)	11 (12.5%)	12 (9.9%)	0.355
Fibrillary	5 (2.4%)	2 (2.3%)	3 (2.5%)	0.647
Renal amyloidosis	4 (1.9%)	3 (3.4%)	1 (0.8%)	0.202

**Total **	209 (100%)	88 (42.1%)	121 (57.9%)	

^*∗*^Statistically significant difference. FSGS: focal and segmental glomerulosclerosis; MGN: membranous glomerulonephritis; IgAN: IgA nephropathy; MPGN: membranoproliferative glomerulonephritis; CGN: crescentic glomerulonephritis; MCD: minimal change disease; MesPGN: mesangioproliferative glomerulonephritis; GN: glomerulonephritis.

**Table 2:** Distribution of glomerular diseases based on etiology.

**Diagnosis / Gender **	**Primary Glomerulonephritis**	**Secondary Glomerulonephritis**	**P value**
**Female No./ (**%**)**	45 (42.1%)	76 (74.5%)	< .001^*∗*^
**Male No. /(**%**)**	62 (57.9%)	26 (25.5%)	

**TOTAL**	107 (51.2%)	102 (48.8%)	

^*∗*^Statistically significant difference.

**Table 3:** Distribution of glomerular diseases based on patients' age.

Diagnosis	Adolescent ≤18 years (%)	Adult 19–59 years (%)	Elderly ≥ 60 years (%)
Primary Glomerulonephritis			

MGN	2 (8.7%)	27 (15.8%)	3 (20.0%)
IgAN	1 (4.3%)	16 (9.4%)	0 (0%)
FSGS	3 (13.0%)	9 (5.3%)	0 (0%)
MCD	4 (17.4%)	6 (3.5%)	0 (0%)
MesPGN	2 (8.7%)	7 (4.1%)	0 (0%)
MPGN	1 (4.3%)	8 (4.7%)	0 (0%)
Chronic GN	0 (0%)	8 (4.7%)	1 (6.7%)
Post infectious GN	0 (0%)	4 (2.3%)	0 (0%)
Alport disease	0 (0%)	1 (0.58%0	0 (0%)
CGN	0 (0%)	4 (2.3%)	0 (0%)

Secondary Glomerulonephritis			

Lupus nephritis	10 (43%)	58 (33.9%)	2 (13.3%)
Diabetic nephropathy	0 (0%)	17 (9.9%)	6 (40%)
Fibrillary	0 (0%)	4 (2.3%)	1 (6.7%)
Renal amyloidosis	0 (0%)	2 (1.2%)	2 (13.3%)

Total	23 (11.0%)	171 (81.8%)	15 (7.2%)

## Data Availability

The pathological reports of the 209 patients with GN-diagnosed biopsies and their medical records were available at the university with the author. This study was conducted in accordance with the World Medical Association Declaration of Helsinki principles and ethical approval was obtained as per the university hospital protocols.
